# Use of a focus group-based cognitive interview methodology to validate a cooking behavior survey among African-American adults

**DOI:** 10.3389/fnut.2022.1000258

**Published:** 2022-12-05

**Authors:** Nicole Farmer, Tiffany M. Powell-Wiley, Kimberly R. Middleton, Alyssa T. Brooks, Valerie Mitchell, Melissa Troncoso, Joniqua Ceasar, Sophie E. Claudel, Marcus R. Andrews, Narjis Kazmi, Allan Johnson, Gwenyth R. Wallen

**Affiliations:** ^1^Translational Biobehavioral and Health Disparities Branch, National Institutes of Health Clinical Center, Bethesda, MD, United States; ^2^Social Determinants of Obesity and Cardiovascular Risk Laboratory, National Heart, Lung, and Blood Institute, National Institutes of Health, Bethesda, MD, United States; ^3^Intramural Research Program, National Institute on Minority Health and Health Disparities, Bethesda, MD, United States; ^4^Daniel K. Inouye Graduate School of Nursing, Uniformed Services University of the Health Sciences, Bethesda, MD, United States; ^5^Department of Nutritional Sciences, Division of Allied Health Sciences, College of Nursing and Allied Health Sciences, Howard University, Washington, DC, United States

**Keywords:** cognitive interview (CI), cooking survey, African-Americans, survey development, dietary behavior assessment

## Abstract

Disparities in diet-related diseases persist among African-Americans despite advances in risk factor identification and evidence-based management strategies. Cooking is a dietary behavior linked to improved dietary quality and cardiometabolic health outcomes. However, epidemiologic studies suggest that African-American adults report a lower frequency of cooking at home when compared to other racial groups, despite reporting on average cooking time. To better understand cooking behavior among African-Americans and reported disparities in behavior, we sought to develop a survey instrument using focus group-based cognitive interviews, a pretesting method that provides insights into a survey respondent’s interpretation and mental processing of survey questions. A comprised survey instrument was developed based on input from a community advisory board, a literature review, and a content review by cooking behavior experts. The cognitive interview pretesting of the instrument involved African-American adults (*n* = 11) at risk for cardiovascular disease who were recruited from a community-based participatory research study in Washington, D.C., to participate in a focus group-based cognitive interview. Cognitive interview methodologies included the verbal think-aloud protocol and the use of retrospective probes. Thematic analysis and evaluation of verbalized cognitive processes were conducted using verbatim transcripts. Five thematic themes related to the survey were generated: (1) Clarity and relevancy of question items; (2) influence of participants’ perspectives and gender roles; (3) participant social desirability response to questions; (4) concern regarding question intent. Eleven survey items were determined as difficult by participants. Cooking topics for these items were: cooking practices, cooking skills, cooking perception (how one defines cooking), food shopping skills, and socialization around cooking. Question comprehension and interpreting response selections were the most common problems identified. Cognitive interviews are useful for cooking research as they can evaluate survey questions to determine if the meaning of the question as intended by the researcher is communicated to the respondents—specific implications from the results that apply to cooking research include revising questions on cooking practice and skills. Focus-group-based cognitive interviews may provide a feasible method to develop culturally grounded survey instruments to help understand disparities in behavior for culturally relevant diet behaviors such as cooking.

## Introduction

Disparities in diet-related diseases persist among African-Americans despite advances in risk factor identification and evidence-based management strategies (1). Bridging the gap in diet-related disease disparities may require attention to more proximal causes of diet-related diseases that lower these risk factors. These proximal factors include lifestyle-based ones, such as dietary behaviors. Among dietary behaviors, a focus on cooking and meal preparation is of interest as several studies show a positive association between cooking and improved dietary quality regarding lower content of daily calories (2), sodium, high fiber (3), and vegetable intake (4), including among African-American adults (5). The community-based participatory research (CBPR) interventions designed to promote cooking, and healthy eating in African-American communities, particularly among adults with an increased risk of cardiovascular disease, have reported improved self-reported dietary quality (6, 7). However, on average, African-Americans report lower home cooking frequency than other racial/ethnic groups in U.S. population-level surveys (8–10), with African-American men reporting the lowest cooking activities among all racial/ethnic groups (10). Investigations into the reason for this disparity in self-reported home cooking have been limited and consist mostly of analyses based on weekly or prior 24-h interview data (5, 9, 10). Although limited, these analyses do point to a need for closer evaluation. For example, from 2014 to 2017, among surveyed ethnic groups in the American Time Use Survey (ATUS), African Americans had the lowest percentage of activity occurrences in preparing food but, on average, did not have the lowest amount of time spent on preparing food. This discrepancy between self-reported engagement and the time spent on food preparation suggests that more than expected time investment may be considered among African Americans when meal preparation occurs. Thus, a key area of research needed is to understand the explanatory reasons for discordances between lower cooking frequency on average juxtaposed with an ethnic, cultural connection to cooking, as represented by the average longer time spent cooking.

The use of surveys to capture dietary behaviors is a well-established methodology. The advantage of surveys is that they can be used for population-level evaluation to gather large amounts of data, offer ease of administration concerning costs, and provide the potential for reproducible findings. Thus, surveys provide a significant source of evidence for nutrition research. And when sampling design and size are considered, well-designed surveys can provide valid and reliable information. To date, the use of surveys to evaluate cooking behaviors among African-Americans has been limited to one study conducted by Condrasky et al., in which a comprised survey instrument was validated among the African-American faith-based cohort (11). The survey measured self-efficacy, cooking knowledge, and attitudes toward cooking but did not include other topics known to contribute to cooking behaviors, such as food and cooking skills, frequency of home cooking, and socialization among social groups regarding cooking. To our knowledge, surveys including these topics have only been validated among non-African-Americans adults (12–14).

One limitation of surveys is the inability to determine how the respondent interprets and cognitively processes the questions and responses (15). Cooking is a behavior that is culture-specific in that practices and behaviors are contextualized by lived experiences, family influences, daily routines, identity, socioeconomic position, and geographic location (16–18). In the context of measuring a culturally specific behavior, not understanding how respondents interpret surveys could threaten the validity of survey results. This limitation may be minimized, however, through the use of pretesting methodologies. In general, pretesting consists of both qualitative and quantitative techniques and activities used to evaluate survey instruments before data collection begins (19). It may involve piloting a survey tool, cognitive interviewing, or focus groups to inform researchers about specific aspects of a tool, such as question wording or length of the instrument. Pretesting also allows for the potential reduction of measurement error (15).

Wallen et al. suggest that the inclusion of cognitive interviews in the initial pretesting of a survey tool may assess how participants interpret and process survey questions based on their own experiences (20, 21). Notably, for survey development, cognitive interviews and interpretation assessment based on experiences could be a beneficial pretesting method because responding to survey questions involves multiple cognitive processes resulting from the utilization of long-term and short-term memory (22). However, cognitive interviews are often considered in large-scale survey evaluations. The utilization of cognitive interviews in small-scale community-based studies has reported an improvement in assessing informed judgments and responses from participants (23, 24). Cognitive interviewing may be valuable for culturally specific topics as the interpretation of questions can vary across cultural experiences (25). There is an emphasis on not only the individualistic mental processing of survey items but also on the background social and environmental context that influences how well questions “meaningfully capture the life of the respondent” (26).

There is an emerging topic in the field of cooking research regarding the lack of and limited scope of cooking behavior metrics and tools. One recent systematic review of cooking measurement tools found that a limited number of studies adequately report the reliability, content, and construct validity of cooking measurement tools (19, 27, 28). Currently, survey instruments utilized during cooking interventions predominately include pre-and post-intervention assessments of cooking confidence, efficacy, and dietary intake (29). Nevertheless, cooking is a complex behavior involving the integration of cognitive, perceptual, and mechanical skills (30), as well as social influences (31). Cooking is also a culturally specific behavior. Thus, pretesting of a survey could allow for the evaluation of cultural appropriateness, such as the target population’s familiarity with constructs of interest in the survey or the use of culturally relevant culinary terminology (19). In particular, attention should be paid to the lack of cultural diversity in the development of cooking survey measurements, as most survey instruments are developed based on European views of cooking methods and practices (28). For example, Bernado et al. showed that although an intervention led to an increase in self-reported dietary intake and cooking confidence compared to the control group, the intervention did not lead to an increase in the reporting of cooking practices at home, as measured by standard survey tools. The authors hypothesized that cultural interpretation of cooking practice and behavior by their Brazilian sample may have contributed to the null result (32). Relevant to cultural adaptation, only two studies in the aforementioned review included cross-cultural adaptation, implying a need for attention to cultural adaptation and significance when developing surveys to assess cooking behavior and to evaluate cooking interventions.

The purpose of this manuscript is to describe the cognitive interview pre-testing of a cooking behavior survey as a part of study development in planning a cooking intervention involving African-American adults at risk for cardiovascular disease (33). We utilized the methodology of cognitive interviewing using a focus group of African-American adults at risk for cardiovascular disease to pretest the survey and to explore the use of cognitive processes to develop a community and culturally relevant cooking behavior measurement tool.

## Materials and methods

### Community-based development

**FIGURE 1:**
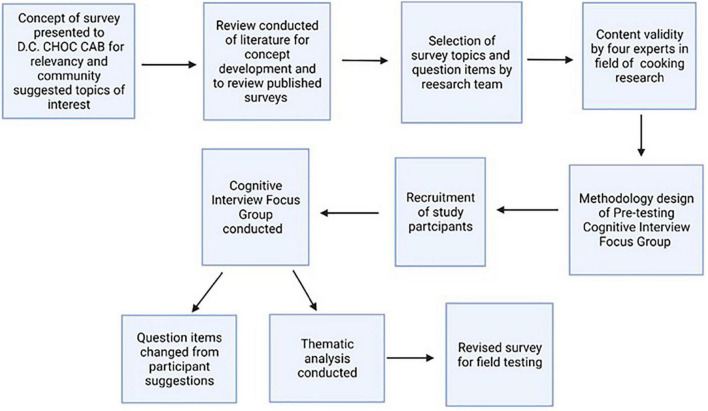
Schema of survey instrument development. Survey instrument development using cognitive focus group pretesting design. D.C. CHOC, D.C. Cardiovascular Health and Obesity Collaborative; CAB, community advisory board.

The formative survey validation pretesting research presented in this paper is part of a long-term community participatory research study within the Washington, DC, metropolitan area exploring cardiovascular risk factors among African-Americans and designing culturally specific, community-based interventions to address these risk factors. The *D.C. Cardiovascular Health and Obesity Collaborative* (D.C. CHOC) was established in 2012 and is made up of our multidisciplinary research group (including physicians, health behaviorists, mixed methodologists, epidemiologists, and research fellows), university faculty in nutrition and community health, and church leaders from predominantly African-American, faith-based organizations in Washington, D.C, wards 5, 7, and 8; areas of the city with the highest CVD prevalence and where access to physical activity resources and healthy nutrition is limited (34). *The Washington D.C. Cardiovascular Health and Needs Assessment* (NCT 01927783) was the first research study designed by D.C. CHOC (35). One of the overarching goals of the study was to assess potential psychosocial and environmental barriers to behavior change concerning physical activity and a healthy diet. *The Washington D.C. Cardiovascular Health and Needs Assessment project* included the evaluation of focus group cognitive interviewing as a method for survey testing, as approved by the National Heart, Lung, and Blood Institute Institutional Review Board (NCT# 01927783). Specific details of *The Washington D.C. Cardiovascular Health and Needs Assessment* can be found in prior publications from our group (35–37). The survey development study proposal and concept were presented to D.C. CHOC and perceived as relevant to the community’s interest in limited dietary choices and the community interest in intervention studies that might help with dietary behaviors among African-Americans living in the Washington, DC, metropolitan area. Additionally, in prior focus groups with this sample population, participants emphasized a desire to identify and participate in community cooking groups (38). The survey development study proposal was approved by the Intramural Research Program NIH Institutional Review Board (IRB) as an amendment to *the Washington D.C. Cardiovascular Health and Needs Assessment*. The development of the current survey instrument included multiple steps, starting with community-based development of topics and assessment of relevancy. Figure 1 provides an overview schema for the survey instrument development methods.

### Recruitment of focus group participants

Using purposive sampling, we recruited a sub-sample of self-reported African-American individuals who were proficient in English and had previously participated in *The Washington D.C. Cardiovascular Health and Needs Assessment* study (*n* = 99, mean age 59.1 years). Prior studies show that marriage is a behavioral factor in cooking. Specific to African-American adults, marriage may be a more positive factor for women than for men. To ensure representation within the focus group from individuals who cook frequently and infrequently, as well as to ensure a significant number of men within the group, the recruitment of married couples was preferred for the focus group. The *Health and Needs Assessment* study coordinator contacted participants by phone and email.

### Development of survey items

A narrative review of peer-reviewed published literature regarding cooking behavior (including cooking skills and cooking confidence) was conducted to determine the constructs (topics) for our survey, including psychosocial factors related to cooking among African-American and non-African-American populations in the peer-reviewed literature. A result of the review used two broad categories for constructs for the survey development: intrapersonal, interpersonal, and cooking practice (frequency). Intrapersonal constructs identified were cooking confidence and self-efficacy (39); attitudes and beliefs, including cooking identity (40), neophilia (the interest in trying new foods), cooking perception (41); and skills, including food skills (42) and cooking skills (42). Condrasky et al. (6) developed a composite survey tool. They conducted face and content validity with experts and test/retest reliability with members of CBPR studies in the Cooking with a Chef program and the faith-based cooks training for faith, activity, and nutrition project, from the same Southern U.S. locality (6, 11). The survey tool by Condrasky et al. identified cooking attitude and self-efficacy for cooking as factors in determining participation in-home cooking pre- and post-intervention (6, 39). Items from the tool developed by Condrasky et al. were previously described by Michaud (39). To limit response bias to questions regarding confidence with cooking techniques and methods, items for this topic were given specific instructions to only rate confidence in cooking skills that respondents have practical experience with, as described by McGowan et al. (31). This avoids participants rating their confidence in skills that they have no experience doing. Cooking skills, mechanical, perceptive, academic, and cognitive, were defined based on home cooking ethnographic studies by Short, F (30) and question items derived based on descriptions by Ternier (43).

Interpersonal constructs identified from the literature included socialization around cooking, social group influence during the life course, and current-day experience with cooking, as described by Urdapilleta et al. (44). These constructs also reflected interpersonal concepts of interest reported to the research team from the D.C. CHOC CAB. Cooking practice questions represented the frequency per week of cooking dinner at home, and the time for preparation of meals was sourced from the Flexible Consumer Behavior Survey used in the 2007–2008 and 2009–2010 National Health and Nutrition Examination Surveys (NHANES) (45, 46). Previous cognitive interviewing by the National Center for Health Statistics on this question showed that cooking practices such as batch cooking might be necessary when asking about home cooking frequency (47). Food security questions were sourced from the USDA Food Security Question/Screener (48). Supplementary Table 1 provides survey topics cross-referenced with determinants for cooking reported in the literature and referenced sources.

The comprised survey consisted of 28 closed-ended questions, with seven questions having multiple sub-sections, leading to a total of 96 question items. Any revisions to a previously published question were due to content clarification or to shorten the question with no intent to change the objective of the question. Before pretesting, the survey topics and items were reviewed and agreed upon by four external experts selected by the lead co-author (NF) through professional organizations on nutrition and a literature review on cooking interventions. Two experts held expertise in family nutrition interventions in diverse communities, and two were clinical dieticians. None of the experts were members of the research team.

### Pretesting: Focus group-based cognitive interview method

When conducting qualitative research, the researchers operate as the instruments for analysis, making judgments about coding, theming, decontextualizing, and recontextualizing the data (49). Thus, it is important to state the epistemological and ontological contexts in which the researchers approached the study and data analysis. For this study, a pragmatism-based paradigm was used from the understanding that an individual’s connection with cooking in their lived experiences may be based on everchanging knowledge and may be best measured using multiple methodologies. Pragmatism is based on the proposition that researchers should use the philosophical and/or methodological approach that best corresponds with the research problem being investigated (50). Pragmatism is often associated with the use of multiple methods. Our study used the combined methodologies of focus groups and cognitive interviewing to conduct *focus group-based cognitive interviewing.* This methodology combines cognitive interviewing within a focus group methodology and a pretesting methodology to further validate the cooking behavior survey.

Although historically, cognitive interviews are conducted *via* individual face-to-face interviews, focus group-based cognitive interviews are reported in the literature for survey development (51–53). There are several reasons why focus groups may be advantageous. The open-ended dialogue could be promoted because focus groups are reported as akin to communicative events in which participants draw on background knowledge acquired through past experiences. This may allow a focus group participant to infer what was intended based on cultural norms, including familiar terminology. Sharing perspectives through past experiences can assist in creating accountability between researchers and study participants and a shared understanding of the research process (54). This understanding of past experiences is particularly important in the context of community-based participatory research (CBPR). Focus groups are noted as a valid method that can provide insights into a target population’s context and cognitive processes (55). In fact, we and others have found that cognitive-based focus groups can decrease the facilitator’s burden and promote open-ended dialogue among participants that would not have been facilitated in a one-on-one interview (54). Our own work conducted within the larger community-based study among African-American adults and work by Crowley et al. showed that cognitive-based focus groups yielded richer data when compared to cognitive-based individual interviews (54).

**FIGURE 2:**
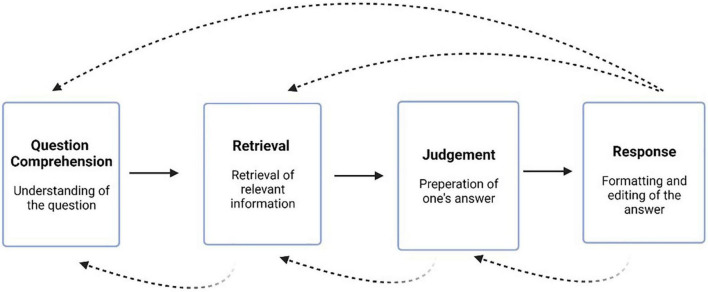
Cognitive processing steps: comprehension, retrieval, judgment, and response. Solid arrows show a linear forward direction between processes. Dotted arrows show potential recursive processes between steps (15).

The methodology of cognitive interviewing allows a researcher to assess the four steps involved in answering survey questions (Figure 2): comprehension (understanding of the question), retrieval (retrieving relevant information), judgment (preparing one’s answer), and response (formatting and editing an answer) (15). Concurrent and retrospective probing cognitive interview strategies and think-aloud strategies may be utilized to elicit a respondent’s understanding of a question (21). Think-aloud strategies encourage participants to verbalize their thoughts by answering cognitive-based questions about survey items (56). One example is “try to visualize the place where you live and think about how many windows there are in that place. As you count the windows, tell me what you are seeing and thinking about” (56).

When verbalizing an individual’s thoughts, a key assumption is that working memory, which includes both short-term and long-term memory processes, is being engaged, allowing individuals to report what they are actively thinking (57). Thus, the verbal report that the respondent is asked to do in cognitive interviews helps to gain insight into the cognitive processes taking place. Upon answering, participant responses may then be placed into one of the aforementioned cognitive steps (15).

In addition to think-aloud strategies, the use of verbal probes may also be employed during cognitive interviews, either in conjunction with think-aloud strategies or as an alternative (58). Probes may be concurrently administered during the cognitive interview or retrospectively immediately following the cognitive interview. Either concurrent or retrospective probes may be standardized (predetermined) or spontaneous (51–53).

### Data collection

A moderator-led focus group at one of the D.C. CHOC collaborating centers, a local university, occurred in early November of 2017. Written informed consent was obtained from all study participants on the day of the focus group. Focus group participants (*n* = 11) were provided a printed copy of the self-administered cooking behavior survey to complete independently. Participants were provided instructions before starting the survey. The instructions involved asking the participants to answer the question items individually and then rate each item as “easy” or “difficult.” Participants were asked to make notes next to each item, including rewording suggestions, adding words/terms, or identifying errors. The start and completion times for each participant were recorded on each survey. Once all participants completed their survey and independent ratings of each question, a brief break was taken before starting the cognitive-based questioning.

The cognitive-based focus group was conducted using a semi-structured moderator guide (Supplementary Figure 1). An experienced moderator (GW) co-led the focused discussion with a co-moderator (NF) trained in cognitive interview techniques (11). Before the focus group, the moderator and co-moderator had no previous relationships with the focus group participants. During the cognitive-based focus group, the survey was reviewed in sections by the survey topics. Before the cognitive-based questioning, participants were asked to perform a “think-aloud” exercise to practice their ability to answer the cognitive-based interview questions (Table 1). For each survey section, a page-by-page and question-by-question review of the survey occurred in which participants were asked if they identified any questions as “difficult.” Identified “difficult” questions on a page were discussed first using the moderator’s cognitive-based guide questions to find out what they understood by each item/statement and if they thought the questions were clearly stated. Participants were then asked if there were questions on a page labeled as “easy” that required rephrasing or rewording. If “easy” questions were identified as such, participants were asked cognitive-based interview questions about those items. The focus group participants were permitted to refer to their completed printed survey and contemporaneous notes during the focus group. The moderator only moved to the next survey section when participants identified no further recommendations for that section.

**TABLE 1:** Examples of verbal probes utilized during the cognitive interview focus group during pretesting of the cooking behavior survey.

Verbal probe type	Illustrative verbal probes utilized	

	Pre-planned	Spontaneous
Comprehensive probe	What do you think the question is asking?	When you saw that question, did you start thinking about activities (*Participant response: “Right.”*) that you do while the food is on?
Paraphrasing probe	None utilized due to probe requiring respondent comment.	Did you mean that we should separate the types of meals in the question?
Recall probe	What type of information did you need to recall (remember) to answer the question?	And when you saw the question did you think just about preparation or did you put all of that together?
Specific probe(s)	Any questions that were difficult from this page of the survey?	For choice “a” what came in your mind was what components of your meal come from scratch? (P: Yes.) As opposed to the whole meal coming from scratch? Did anyone else have that thought too as they went through question eight?
General probe	Tell me what are you thinking?	What were you thinking when you were answering that question and then you didn’t have the category available?

Once all sections of the survey were reviewed, and the cognitive-based questioning was concluded, participants were allowed to relate their reasoning for question item changes to their lived experiences. During this part of the focus group, probes were utilized to elicit further responses (Table 1). Probing questions were proactive (researcher predetermined) and reactive probes (based on respondent answers). All questions were retrospectively asked after participants completed the survey. The moderator additionally asked participants if there were question items that did not pertain to their lived experiences and why that required rephrasing, contained errors, or elicited comments based on the participant’s lived experiences with cooking at home. Throughout the moderation of the focus group, the moderator summarized statements to provide participants with opportunities to clarify their responses. Two other team members operated the audio recorder and took written field notes. Field notes were used to record non-verbal or informal verbal communication. The total focus group was concluded when participants identified no new topics for discussion. The total focus group lasted 96 min, including the cognitive-based questioning.

### Data analysis

The focus group was audio-recorded, with the recording transcribed verbatim into anonymized transcripts by an independent clinical research company. A member of the research team (NF), who conducts research primarily on cooking behavior and measurement tools, verified the quality of the verbatim transcripts from the audio recordings. Before the start of the thematic analysis, the research team determined that both an inductive and a deductive approach to the data would be employed. This determination was made through an initial review of the transcript by members of the research team who independently reviewed the transcripts and field notes and then met collectively to discuss the transcript and respective notes. The deductive approach consisted of evaluating transcripts using the four steps of the cognitive process: comprehension, retrieval, judgment, and response. For the inductive approach, quotes were coded and categorized into themes and subthemes, as described in Braun and Clarke (59). The inductive approach was guided by a phenomenological approach to the data to capture the participant’s lived experiences in relation to survey questions or constructs. Thematic analysis of the verbatim interview transcript was done by three members of the broader three research teams (NF, NK, and MT). All three researchers involved in this step were experienced in community-based research and qualitative data analysis. Each coder independently read the transcript, conducted coding, and wrote reflective notes. The further steps of the process occurred during in-person meetings among the coders. These steps included creating a thematic framework, comparing notes among coders, and further defining and describing the themes. Next, the coders evaluated the transcript for connections across themes and data saturation. During the meetings, all coders offered critical feedback on interpretations. The process considered other coders’ interpretations and the time and ability to reflect on others’ perspectives (59, 60). All coders participated in the write-up of the analysis and theme review.

Once the consensus-building process was complete, a team member experienced in qualitative data analysis (GW), not previously involved with coding quotes into themes, validated the themes and codes.

To identify the potential influential role of probes in eliciting participant responses, focus group transcripts were reviewed to evaluate the use and type of retrospective prompts by moderators as described in Park et al. (58). Table 1 provides the types of probes utilized during the focus group. During the cognitive-based questioning, comprehensive and recall probes were used once a participant identified a question to discuss and verbalized their response to the think-aloud instruction when first discussing the item. These two probes were used to identify additional information the participant may have needed to answer the item and provided insight to the researchers regarding the cognitive steps used to provide a response. The only probe used before the think-aloud step was the specific probe to identify difficult questions. When needed, a general probe was used to elicit a follow-up response (i.e., “When you answered that question and didn’t have a category that reflected your experience, what did you think about that?”).

The trustworthiness of the data was measured using the tenets of credibility, transferability, dependability, and confirmability. Credibility was ensured through the use of intermitted peer briefings with non-coding members of the research team. Our use of purposive sampling addressed transferability. The coders created an audit trail from in-person meetings throughout the coding process and maintained reflexive notes representing dependability and confirmability, respectively.

Following the qualitative data analysis, changes to the survey tool that reflected the focus group findings were discussed by two co-authors, the lead author (NF) and a trained survey methodologist (KM). NVivo 9.0 (QSR International Pty Ltd, Melbourne, VIC, Australia) was used to organize and query the qualitative data. Socioeconomic and demographic data were summarized using IBM SPSS Statistics version 24 software (SPSS, Chicago, IL, USA) (61).

## Results

Focus group participants (*n* = 11) were primarily female (*n* = 7), with an average age of 65 (SD 8.5) years old (Table 2). Nine participants in the focus group were married, and eight were married couples within the focus group. As an illustration of cooking and cooking behavior, the reported cooking frequency of dinner was a mean of 3.3 days per week (SD 1.6).

**TABLE 2:** Demographic and cooking frequency data for focus group members, *n* = 11.

Characteristics	Values
**Age, years**	
Mean (SD)	65 (8.5)
Range	51–75
Gender	No. (%)
Female	7 (63.6)
Male	4 (36.3)
**Race**	
Black/African-American	11 (100)
**Marital status**	
Single	1 (9.1)
Married	9 (81.8)
Divorced	1 (9.1)
**Education**	
High school grad or GED	1 (9.1)
Some college	4 (36.3)
College degree	3 (27.3)
Graduate/professional degree	3 (27.3)
**Location of residence**	
Maryland (Metropolitan D.C. area)	6 (54.5)
Washington, D.C.	5 (45.4)
Annual household income by metropolitan area median income^a^*	
<$93,294	8 (80)
>$93,294	2 (20)
Mean cooking frequency/week (SD)	3.3 (1.6)

^a^2015 US Census Bureau median household income for District of Columbia metropolitan area.

*One respondent did not answer the income question.

As discussed in the Section “Materials and methods,” focus group participants were instructed to identify items that were difficult to read, understand, or answer. Focus group participants identified 11 items from the total survey as “difficult.” These items represented questions from four topics: cooking practices, cooking skills, cooking perception, and social/development exposure to cooking (Supplementary Table 2). Each “difficult” item mapped to one of four cognitive processes: comprehension of the question, recall of requested information from memory, judgment evaluation of the link between the retrieved information and the question, and the communication of the response (Supplementary Table 2).

### Themes

Thematic analysis of the focus group transcript yielded four themes: clarity and relevance of items, the influence of participant perspectives and gender roles, participant responses to questions, and concerns regarding the reason behind the intent. These themes contributed to survey development through question item changes to reflect and capture the discussion put forth by the participants. The revised survey is available in Supplementary Figure 2. Discussion of each theme/subthemes, illustrative quotes from participants, and the resultant survey development change are detailed below under each theme.

#### Theme 1: Clarity and relevancy of question items

The clarity of question items was discussed by participants, particularly for questions pertaining to cooking practices, such as who makes meals at home and time spent on cooking. In response to the survey question, “Which person in your household most often prepares meals,” a 63-year-old married female reported, “*Sometimes my husband does the breakfast and I do the dinner. So, maybe you should separate that and say breakfast and dinner*…” Confirming her statement, a 66-year-olf-female stated, “*That’s true for us too. He does the breakfast.*”

With regard to time, several participants expressed that questions asking about time spent cooking should clarify time exclusively for cooking or time in which simultaneous other household activities are occurring. For example, a 67-year-old female stated the following:

“How do you figure out the time it took you to cook cause you’re doing so many other things. So, it’s hard for you to say it took me an hour or… because then I’m like they would have put something on and then run upstairs and get laundry, run back downstairs. It’s not like I’m just sitting there in the kitchen.”

Another participant added in response:

“And I think also sometimes it can depend on if you’re using a recipe, about how the recipe you have to marinade. So you could take into consideration the night you might marinate the meat and then the next day you might cook it and then the next day you might cook the sides.”; 55-year-old female

The last statement by the respondent led to a discussion regarding when people choose to prepare foods based on their home life schedules.

“What I had to learn to do was prepare my meals from before and when I come home that only the vegetable and maybe the starch or whatever the carbohydrates that I would have, that I would have to cook. So, even though I didn’t like having to cook my meats way before, it offered a way that it made it easier because I mean when you’re going to school, and you’re coming home at seven o’clock”; 61-year-old female “That’s why I suggested earlier, because of convenience sometimes I would go to Safeway or Costco or wherever and get my meat and then since last night I made rice, brown rice it’s the healthier choice and I cook that to have for a few days.”; 55-year-old female

The survey development change from this theme provided specification of a respondent’s time participating in cooking actively to reflect possible competing activities during meal preparation, and the addition of a direct question to characterize if participants batch cooked as a part of their foodwork strategy for time availability.

The relevancy of specific question items to participants’ food shopping and cooking behaviors was identified. For the question about shopping for “cheaper cuts of meat,” several participants responded with suggestions for additions to the question to reflect different preferences:

“And at the end of the day, a lot of our meats now people are going to the organic side. Farm-raised opposed to the way…they better raising them, which means it’s going to be a higher cost for people as well. So, when you start doing your survey now, you may want to include the difference with buying organic and buying farm the way they’re what do they call that?”; 61 year-old female.

Pertaining to both the need for clarity and relevance to how participants’ used kitchen equipment for the cooking skill of cutting vegetables, participants discussed the specific term “knife skills” used in question items on cooking confidence. In reference to the question asking if a participant used knife skills to cut vegetables, a 61-year-old-female stated that:

“For that one, I answered I do not at all because I was associating what they were saying with the (participant makes quick thumping sound to simulate knife on cutting board) right, because I don’t want to lose my fingertips so I don’t do that (chop). But I do cut up and so I would clarify the question with not chopping”.

In agreement with the interpretation of knife skills, one 63-year-old female also stated the following:

“And so when you hand hold the green pepper in your hand and you’re slicing I would consider that more cutting up rather than (participant makes quick thumping sound) And that’s why I said I do not at all but I clearly cut up vegetables.”

Geography was mentioned as influential to this same participant about growing up “down South,” and accessibility to kitchen equipment. A 55-year-old female stated

“When I grew up, we didn’t have a cutting board so we did it by hand so now we have cutting boards but when I was growing up down South we didn’t have a cutting board.”

The resultant survey change was to clarify the term knife skills to include the use of kitchen tools to chop or cut food during cooking.

Lastly, the relevance of the neighborhood context was expressed by some participants when questions were asked about food shopping. For instance, one participant stated

“So, therefore it might not be it’s not available and now they have what’s called corner stores and they’re supposed to have healthy food in these corner stores but before you get to the healthy foods you go past the potato chips, the soda…and then the healthy it’s back in the corner and it looks like crap”; 67-year-old female.

#### Theme 2: Influence of participants’ perspectives and gender roles

Some male participants reported difficulty with answering questions related to cooking practices at home.

For the question asking “who does most of the cooking in the home,” one female respondent answered while referring to her husband, “*He does the breakfast*,” a 66-year-old female. This response was agreed upon by the group of participants that meal-specific cooking occurs in their home as opposed to all of the cooking occurring by one person.

Related to the time spent cooking, a gender-specific role was expressed, limiting a participant’s ability to answer the question on time spent cooking.

“I said I don’t know, because she does most of the cooking and then same thing with cleaning up after cooking dinner. I thought about maybe half an hour for that but I actually put 45 min”; 77-year-old male.

With respect to the survey changes, the gender discussion led to the removal of the question asking “*who does most of the cooking in the home*,” as this question brought on a discussion about meal-specific roles by gender. And to avoid answers in which a respondent would answer based on their perception of their spouse’s role, a direct question identifying the respondent’s direct cooking activity was added to the survey tool.

#### Theme 3: Participant responses to questions Social desirability

Participants noted a feeling of wanting to select an answer choice for “from scratch foods” over “convenience foods,” as well as hesitation to report the use of deep-frying as a cooking method at home. One 77-year-old female stated

“To be honest I had felt like I needed to do something with a (choice with scratch foods) because I can clearly see that (choice) “a” is better than (choice) b and (choice) c (choices b and c were choices with convenience foods).”

Regarding the selection of cooking methods, a 51-year-old male reported vacillating opinions about his response to a question regarding cooking confidence because the question asked to rate confidence in a skill or method that a person has practical experience doing. For the use of deep frying, the respondent stated, *“That’s a guilty question. Even though we don’t, I know we do.”* The survey was changed from asking participants to choose an affirmative response of “I do” for techniques. Instead, the survey question emphasized that if a technique is done at home for, participant to mark their confidence level with the technique.

#### Theme 4: Concern regarding question intent

Two questions elicited an inquiry response by participants regarding the researchers’ intent to use terms in the question that included cheaper. “*The only thing that came up for me was what was driving this line of questioning about not having enough money or not having enough food or being able to afford it. I was just wondering what was driving this*”; 61-year-old female. And *“I have a question for g (cheaper meats choice), on top of the list. Buying cheaper cuts of meat to save money. I would also put under you can buy better carved meat that’s on sale,”* 55-year-old female.

The original survey was revised in response to the coding of themes and cognitive problems. Changes to ‘difficult’ items are presented in Supplementary Table 2. Other revisions to the survey included the addition of an item to identify if the respondent was indeed the person who cooked at home, adding an item of self-reported diet quality, and adding social media and the internet as cooking recipe sources. The revised survey is provided in Supplementary Figure 2. Following the revision to the survey, field-testing and evaluating psychometric properties are the next steps for further development of the survey instrument (40).

## Discussion

When used in a study’s formative development phase, cognitive interview approaches can provide valuable insight into subjects’ processes and information needs and inform the development of more effective questions (20, 62). More effective questions allow researchers to not only collect valid data that allows us to make inferences about dietary behaviors but, in the instance of cooking, allows us to make inferences about behavior that can allow for the effective use of surveys in intervention studies. This step in survey development may also help to inform cooking behavior questions in epidemiologic studies (44).

To our knowledge, the combined use of a focus group with a cognitive interviewing approach for survey development has only been reported in one previously published study (21). In our study, the cognitive challenge is most often identified as the response stage (communication of the response). All response stage problems occurred with questions on cooking practice or skills. We found that questions related to perceived ideas and recall of practices did not easily fall into these stages and were often affected by interpretation issues. As observed in other cognitive interview testing reports, some participants found the intent of some questions difficult to comprehend. During the revision of the survey, items were redesigned to have a simple design inquiry, as recommended by Willis and Zahnd (63). For the cognitive problem, judgment and social desirability (64) was a factor in participant responses. As a result, we were able to change the question-wording to take into consideration the possibility of participants offering a socially desirable response instead of their true answer. Interestingly, questions in our survey on cooking self-efficacy and food shopping skills were utilized previously in African-American populations (6) but were identified by participants in this study as requiring changes or clarification. One of those questions using culinary terms such as “knife skills” needed a change due to our focus group’s cultural interpretation of the question. Thus, we suggest that with the use of culinary terminology in a survey, clarification or expansive explanations using cultural definitions of the term be provided. We also observed that participants did not identify any questions related to attitudes and beliefs that were difficult. This may reflect the cited role of semantic memory in answering questions concerning attitudes and behavior (65), whereas episodic memory, which involves more cognitive recall, is involved in answering questions about practices (65).

Our study revealed several topics relevant to the development of cooking behavior surveys. Cooking practices involving frequency, timing, and household member roles in meals, were a topic that required multiple changes. This may be based on community, household, or culture-specific uses of time with regard to cooking, meal planning, and delegation of roles for cooking among household members. Our results on cooking practice also support work by Castelo et al. (66) that cooking is an integrative practice and participation reflects combinations of routines and contexts, such as food, time, location, social setting, mental processes, and physical conditions. Thus, for precise measurement of cooking practices and practice engagement, our results suggest that identifying if cooking is occurring as a primary or secondary activity (i.e., meals cooking while other household activities are occurring) is important to determine. Additionally, structuring answer choices around potential household contexts, such as day of the week, time of the week, in association with other household schedules and activities, and context of family and childcare duties may be a consideration (67).

Relevant to our primary area of interest for the study, the reported disparity in cooking frequency among African-American adults, our results identified participants referencing multiple times that batch cooking or cooking large meals during parts of the week occurred as a part of meal planning. Batch cooking may reflect time-saving strategies and is reported in other qualitative food practice studies among African-Americans (18). This finding led us to add a direct question to the survey to ask if “batch cooking or cooking ahead for weekly meals” occurred in the home. As we found in our focus group-based CI, community-specific concern about stigmatization through question language was discussed. Additionally, presumptions about status and class are inherent to discussions about food and language. In our survey, participants identified questions about status and class as difficult. Having these class-sensitive wordings brought to the researchers’ attention during pretesting was important and led to the rewording of questions. For example, having less emphasis on terms that may indicate class specificity, such as “cheaper,” and inclusion of both “food security” and “food insecurity” as terms, as were implied by our focus group participants. This is an especially important consideration for community-based studies and may not have become evident without a pretesting step and cognitive interview process.

### Strengths and limitations

There are several strengths of our study. Utilization of a focus group allowed for participant interaction, which may provide ease and comfort for discussion and assist in a person’s recall of relevant topics as a result of the group interaction (68). The fact that this study was embedded within a larger CBPR study is another strength, as survey instruments developed within CBPR studies are shown to provide community-relevant results (69). Additionally, CBPR studies help to establish trust among participants and the research team, which may have helped to facilitate discussion based on the participant’s actual concerns, attitudes, beliefs, and lived experiences. Our hybrid cognitive interview methodology of using both think-aloud and retrospective probing represents another strength of our study. Think-aloud protocols suggest a problem with a question, but probes are required to provide information to diagnose the problem with the question item (57). Another strength of our study is that our cognitive interview methodology followed the recommended cognitive interview research framework (70).

There are also limitations of our study that are important to discuss. We utilized a combined methodology of focus groups and cognitive-based interviewing. Although the group setting can provide a forum for participants to feel at ease in their responses, there is also the potential for the group setting to influence participants’ responses. We did not measure the degree to which the group setting may influence responses; thus, we must consider this a limitation of our methodology. Using only one focus group may limit our ability to achieve data saturation. However, work by Guest et al. regarding the number of focus groups necessary for data saturation among a minority population found that although not optimal, one focus group could generate at least a majority of themes among African-American adults (71). In addition, the inclusion of married couples among the focus group participants may have an untoward effect on results or influence the comfort level of participants (68), potentially leading to a reporting bias on behalf of participants. However, it is anticipated that this reporting bias would have led to male counterparts not discussing a desired role in the kitchen or minimally participating in the discussion. Nevertheless, without a non-married comparison focus group, it is difficult to estimate the impact of including married couples in our focus group.

## Conclusion

Determining the impact of cooking behavior on dietary quality and related health outcomes is limited without developing and using reliable, valid, and culturally sensitive survey tools. Our study demonstrated a role for pretesting a cooking behavior survey using a cognitive interview focus group of African-American adults living in the Washington, DC, metropolitan area. Based on our finding of significant changes to our comprised survey tool from focus group suggestions and discussion, conducting pretesting using cognitive interviewing is a useful step in the tool development process. We also identified practical considerations and implications for future survey development on cooking among African-American adults. Future survey development should include the importance of inquiring about food work strategies related to time and household context and the use of inclusive terminology that incorporates multiple tools for food preparation. Key implications for survey development within cooking research are that questions may not be interpreted in the way the author intended, especially for cooking practice and skills questions, use of cognitive interview technique for pretesting may be done in a community setting, and the addition of this step to cooking survey development may strengthen survey design.

## Data availability statement

The raw data supporting the conclusions of this article will be made available by the authors, without undue reservation.

## Ethics statement

The studies involving human participants were reviewed and approved by Institutional Review Board, Intramural Research Program, National Institutes of Health. The patients/participants provided their written informed consent to participate in this study.

## Author contributions

NF, GW, and TP-W contributed to the conceptualization of the manuscript. NF, KM, VM, TP-W, and GW conducted the methodological planning. NF, GW, VM, JC, and AJ conducted the study investigation and including administration. NF, MT, AB, VM, KM, JC, NK, SC, MA, TP-W, and GW performed the formal analysis. NF, NK, MT, KM, and AB conducted the original writing of the draft manuscript. TP-W and GW provided study supervision. All authors conducted the review and editing of the manuscript, contributed to the article, and approved the submitted version.

## Supplementary material

Supplementary Figure 1 Focus group’s moderator guide.

Supplementary Figure 2 Revised survey.

Supplementary Table 1 Survey topics.

Supplementary Table 2 Question items and illustrative cognitive process.
