# Supplementary material for: Use of a focus group-based cognitive interview methodology to validate a cooking behavior survey among African-American adults
Source: Front Nutr. 2022 Dec 5;9:1000258. doi: 10.3389/fnut.2022.1000258 (PMC9760831; doi:10.3389/fnut.2022.1000258)
Supplement: Supplementary Figure 1 — Focus group’s moderator guide. [file Image_1.PDF]

S1 Figure. Moderator Guide.

Question Type	Question wording
**Cognitive Interview Technique**	By 'think aloud' I mean repeating all the questions aloud and telling me what you are thinking as you hear the questions as you pick the answers. Here is an example: visualize the place where you live and think about how many windows there are in that place. When you are counting windows tell me what you are seeing and thinking
**Survey based questions**	On page ____, were there any questions that were difficult to understand? If so, please tell me the question number on the survey that you identify as being "difficult"<br><br>For the questions that you identified as "difficult" or an issue area, could you please tell me why you felt they were difficult?
**Probing questions**	What do you think the question is asking?<br>What do the specific words mean to you?

	What type of information did you need to recall (remember) to answer the question? For example do you recall things individually or do you estimate to answer the question?<br>Do you have to devote mental effort to answer thoughtfully and accurately?
**Additional Moderator instructions**	After each difficult question, please ask the participant for recommendations on modifying/revising the question for the participant's comfort
**Closing question**	I would like to ask you a general question about your cooking practices. What gets in the way of you cooking? Of going shopping for food?
