# Supplementary material for: Use of a focus group-based cognitive interview methodology to validate a cooking behavior survey among African-American adults
Source: Front Nutr. 2022 Dec 5;9:1000258. doi: 10.3389/fnut.2022.1000258 (PMC9760831; doi:10.3389/fnut.2022.1000258)
Supplement: Supplementary Figure 2 — Revised survey. [file Image_2.pdf]

Cooking Survey for Community Based Health and Needs Assessment

Today's Date: ________ **Start time: ______ Stop time: _______**

The questions in this survey will ask about your beliefs and practices surrounding cooking within your home and food shopping. Your answers will help to inform our research team about the design of future nutrition interventions. For each question, please check if you find the question easy or difficult to answer.

The first set of questions are about the time you spend doing different types of cooking activity in a typical week. In answering the following questions, your household is the entire group of persons living in your home; they may or may not be your family members.

1. How would you rate your diet? Make 1st question

- ☐ Excellent
- ☐ Very Good
- ☐ Good
- ☐ Fair
- ☐ Poor

1. Do you cook ? (Combination of old Q 1 and 2)

Assessment Key	
☐ Easy	☐ Difficult

- ☐ Yes
- ☐ No _________,

If your answer is No, please skip to Question 12

2. What days of the week do you cook? Please check all that apply.

☐ Monday ☐ Tuesday ☐ Wednesday

☐ Thursday ☐ Friday ☐ Saturday

☐ Sunday

3. Do you batch cook (make large amounts of food to eat later) meals in advance?
   ☐ Yes ☐ No (*added to replace old Q.3)

4. How many adults and children do you or someone in your household usually prepare food for on a daily basis?

Cooking Survey for Community Based Health and Needs Assessment

Enter number of people ____

☐ Don't know

Assessment Key	
☐ Easy	☐ Difficult

For the next three questions, think about the past 7 days and about your usual or day-to-day routine.

5. During the **past 7 days,** how many times did you (or someone else in your family) cook food for dinner (or supper) at home?

Assessment Key	
☐ Easy	☐ Difficult

☐ 0 ☐ 1 ☐ 2 ☐ 3 ☐ 4 ☐ 5 ☐ 6 ☐ 7

6. How much time do you or someone in your family usually spend on cooking dinner or supper?

   Hours: minutes: ___hrs :____mins

   ☐ Don't know

Assessment Key	
☐ Easy	☐ Difficult

7. How much time do you or someone in your family usually spend on cleaning up after cooking dinner?

   Hours: minutes: ____hrs :____mins

   ☐ Don't know

Assessment Key	
☐ Easy	☐ Difficult

Most people use different ways to prepare meals, the following questions will ask about your day-to-day cooking habits.

8. During the **past month** how often did you do the following?

	Not at all	1-2 times during the past month	Once a week	Several times each week	About everyday
a. Prepare meals from basic "scratch" ingredients, such as whole fresh produce, raw chicken	☐	☐	☐	☐	☐

	Not at all	1-2 times during the past month	Once a week	Several times each week	About everyday
b. Prepare meals using convenience items, such as bagged salad, prepared mashed potatoes, pre-shredded vegetables, deli rotisserie chicken	☐ ☐	☐	☐	☐	☐

	Not at all	1-2 times during the past month	Once a week	Several times each week	About everyday
c. Reheat or use leftovers in another meal	☐	☐	☐	☐	☐

Assessment Key	
☐ Easy	☐ Difficult

9. Merged with Q. 11

Assessment Key	
☐ Easy	☐ Difficult

10. The next group of questions ask about your confidence with different cooking **techniques**.

    Think about which practice/habits you use at home, then please rate your confidence with using the cooking technique.
    If you do **not** do the practice at all at home, please check I do not do at all.

    a. Boiling

    ☐ 1 Not at all Confident ☐ 2 ☐ 3 Slightly ☐ 4 ☐ 5 Moderately ☐ 6 ☐ 7 Very much ☐ 8 ☐ 9 Completely Confident

    ☐ I do not at all

    b. Simmering (staying just below the boiling point when cooking)

Cooking Survey for Community Based Health and Needs Assessment

☐ 1 Not at all Confident ☐ 2 ☐ 3 Slightly ☐ 4 ☐ 5 Moderately ☐ 6 ☐ 7 Very much ☐ 8 ☐ 9 Completely Confident

☐ I do not at all

c. Steaming

☐ 1 Not at all Confident ☐ 2 ☐ 3 Slightly ☐ 4 ☐ 5 Moderately ☐ 6 ☐ 7 Very much ☐ 8 ☐ 9 Completely Confident

☐ I do not at all

d. Deep frying

☐ 1 Not at all Confident ☐ 2 ☐ 3 Slightly ☐ 4 ☐ 5 Moderately ☐ 6 ☐ 7 Very much ☐ 8 ☐ 9 Completely Confident

☐ I do not at all

e. Sauteing (fry quickly in a little hot fat)

☐ 1 Not at all Confident ☐ 2 ☐ 3 Slightly ☐ 4 ☐ 5 Moderately ☐ 6 ☐ 7 Very much ☐ 8 ☐ 9 Completely Confident

☐ I do not at all

g. Stir-frying (frying meat,fish,or vegetables rapidly over a high heat while stirring briskly)

☐ 1 Not at all Confident ☐ 2 ☐ 3 Slightly ☐ 4 ☐ 5 Moderately ☐ 6 ☐ 7 Very much ☐ 8 ☐ 9 Completely Confident

☐ I do not at all

f. Grilling

☐ 1 Not at all Confident ☐ 2 ☐ 3 Slightly ☐ 4 ☐ 5 Moderately ☐ 6 ☐ 7 Very much ☐ 8 ☐ 9 Completely Confident

☐ I do not at all

g. Poaching (staying just below boiling point to cook in a small amount of liquid)

☐ 1 Not at all Confident ☐ 2 ☐ 3 Slightly ☐ 4 ☐ 5 Moderately ☐ 6 ☐ 7 Very much ☐ 8 ☐ 9 Completely Confident

☐ I do not at all

h. Baking

☐ 1 Not at all Confident ☐ 2 ☐ 3 Slightly ☐ 4 ☐ 5 Moderately ☐ 6 ☐ 7 Very much ☐ 8 ☐ 9 Completely Confident

☐ I do not at all

Assessment Key	
☐ Easy	☐ Difficult

11. The next question is regarding your **confidence** with different **cooking practices**.

Think about which practice/habits you use at home, then please rate your confidence with using the cooking practice/habit.
If you do **not** do the practice at all at home, please check I do not do at all.

a. Cooking from basic ingredients (example: whole lettuce heads, fresh tomatoes, raw chicken)

☐ 1 Not at all Confident ☐ 2 ☐ 3 Slightly ☐ 4 ☐ 5 Moderately ☐ 6 ☐ 7 Very much ☐ 8 ☐ 9 Completely Confident

☐ I do not at all

b. Follow a new recipe or cook with new foods (example: preparing fresh salsa from tomatoes, onion, garlic, peppers)

☐ 1 Not at all Confident ☐ 2 ☐ 3 Slightly ☐ 4 ☐ 5 Moderately ☐ 6 ☐ 7 Very much ☐ 8 ☐ 9 Completely Confident

☐ I do not at all

c. How confident are you about adapting recipes to your food preferences or to preferences of others?

☐ 1 Not at all Confident ☐ 2 ☐ 3 Slightly ☐ 4 ☐ 5 Moderately ☐ 6 ☐ 7 Very much ☐ 8 ☐ 9 Completely Confident

☐ I do not at all

d. Use knife skills (chopping) in the kitchen to chop vegetables, not necessarily with a cutting board

☐ 1 Not at all Confident ☐ 2 ☐ 3 Slightly ☐ 4 ☐ 5 Moderately ☐ 6 ☐ 7 Very much ☐ 8 ☐ 9 Completely Confident

☐ I do not at all

e. Prepare and cook fresh or frozen green vegetables

☐ 1 Not at all Confident ☐ 2 ☐ 3 Slightly ☐ 4 ☐ 5 Moderately ☐ 6 ☐ 7 Very much ☐ 8 ☐ 9 Completely Confident

☐ I do not at all

f. Prepare and cook root vegetables (potatoes, beets, sweet potatoes)

☐ 1 Not at all Confident ☐ 2 ☐ 3 Slightly ☐ 4 ☐ 5 Moderately ☐ 6 ☐ 7 Very much ☐ 8 ☐ 9 Completely Confident

☐ I do not at all

g. Prepare and cooking raw meat/poultry

☐ 1 Not at all Confident ☐ 2 ☐ 3 Slightly ☐ 4 ☐ 5 Moderately ☐ 6 ☐ 7 Very much ☐ 8 ☐ 9 Completely Confident

☐ I do not at all

h. Prepare and cook raw fish

☐ 1 Not at all Confident ☐ 2 ☐ 3 Slightly ☐ 4 ☐ 5 Moderately ☐ 6 ☐ 7 Very much ☐ 8 ☐ 9 Completely Confident

☐ I do not at all

i. Make sauces and gravy from scratch

☐ 1 Not at all Confident ☐ 2 ☐ 3 Slightly ☐ 4 ☐ 5 Moderately ☐ 6 ☐ 7 Very much ☐ 8 ☐ 9 Completely Confident

☐ I do not at all

j. Use herbs and spices (fresh or dry) to flavor food

☐ 1 Not at all Confident ☐ 2 ☐ 3 Slightly ☐ 4 ☐ 5 Moderately ☐ 6 ☐ 7 Very much ☐ 8 ☐ 9 Completely Confident

☐ I do not at all

Assessment Key	
☐ Easy	☐ Difficult

12. Below are several different food **shopping practices**, please think about which practice you use when food shopping and rate your confidence. If you do not do the shopping practice asked, please check I do not do all.

a. Shopping with a grocery list

☐ I do not at all

☐ 1 Not at all Confident ☐ 2 ☐ 3 Slightly ☐ 4 ☐ 5 Moderately ☐ 6 ☐ 7 Very much ☐ 8 ☐ 9 Completely Confident

b. Shop for ingredients with specific meals in mind

☐ I do not at all

☐ 1 Not at all Confident ☐ 2 ☐ 3 Slightly ☐ 4 ☐ 5 Moderately ☐ 6 ☐ 7 Very much ☐ 8 ☐ 9 Completely Confident

c. Planning how much food to buy

☐ I do not at all

☐ 1 Not at all Confident ☐ 2 ☐ 3 Slightly ☐ 4 ☐ 5 Moderately ☐ 6 ☐ 7 Very much ☐ 8 ☐ 9 Completely Confident

d. Comparing prices before you buy food

☐ I do not at all

☐ 1 Not at all Confident ☐ 2 ☐ 3 Slightly ☐ 4 ☐ 5 Moderately ☐ 6 ☐ 7 Very much ☐ 8 ☐ 9 Completely Confident

e. Knowing what budget you have to spend on food

☐ I do not at all

☐ 1 Not at all Confident ☐ 2 ☐ 3 Slightly ☐ 4 ☐ 5 Moderately ☐ 6 ☐ 7 Very much ☐ 8 ☐ 9 Completely Confident

f. Buying food in season to save money

☐ I do not at all

☐ 1 Not at all Confident ☐ 2 ☐ 3 Slightly ☐ 4 ☐ 5 Moderately ☐ 6 ☐ 7 Very much ☐ 8 ☐ 9 Completely Confident

g. Buying more economical cuts of meat to save money

☐ I do not at all

☐ 1 Not at all Confident ☐ 2 ☐ 3 Slightly ☐ 4 ☐ 5 Moderately ☐ 6 ☐ 7 Very much ☐ 8 ☐ 9 Completely Confident

h. Using leftovers to create another meal

☐ I do not at all

☐ 1 Not at all Confident ☐ 2 ☐ 3 Slightly ☐ 4 ☐ 5 Moderately ☐ 6 ☐ 7 Very much ☐ 8 ☐ 9 Completely Confident

i. Keeping basic items in your cupboard for putting meals together

☐ I do not at all

☐ 1 Not at all Confident ☐ 2 ☐ 3 Slightly ☐ 4 ☐ 5 Moderately ☐ 6 ☐ 7 Very much ☐ 8 ☐ 9 Completely Confident

j. Reading the storage location, use by date or expiration date on food packets

☐ I do not at all

☐ 1 Not at all Confident ☐ 2 ☐ 3 Slightly ☐ 4 ☐ 5 Moderately ☐ 6 ☐ 7 Very much ☐ 8 ☐ 9 Completely Confident

k. Reading the nutrition information on food labels

☐ I do not at all

☐ 1 Not at all Confident ☐ 2 ☐ 3 Slightly ☐ 4 ☐ 5 Moderately ☐ 6 ☐ 7 Very much ☐ 8 ☐ 9 Completely Confident

l. Balancing meals based on nutrition advice of what is healthy

☐ I do not at all

☐ 1 Not at all Confident ☐ 2 ☐ 3 Slightly ☐ 4 ☐ 5 Moderately ☐ 6 ☐ 7 Very much ☐ 8 ☐ 9 Completely Confident

m. Buying organic foods

Assessment Key	
☐ Easy	☐ Difficult

If you answered that you do not cook in Question 1, then skip to Question 24-28.

13. I can time different parts of a dish to come together at the same time?

☐ Yes ☐ No ☐ Don't know

Assessment Key	
☐ Easy	☐ Difficult

14. I can prepare or cook a meal in a limited amount of time (30 minutes)

☐ Yes ☐ No ☐ Don't know

Assessment Key	
☐ Easy	☐ Difficult

15. I can cook more or double a serving from a recipe to use in a meal

Assessment Key

☐ Yes ☐ No ☐ Don't know

☐ Easy	☐ Difficult

16. I can prepare or cook a healthy meal with only a few ingredients on hand

☐ Yes ☐ No ☐ Don't know

Assessment Key	
☐ Easy	☐ Difficult

17. Everyone's definition of cooking is different. Below are some examples of different ways people prepare meals. Think for a moment about how you define cooking, then indicate if you agree or disagree that the following activities are cooking.

	Disagee	Agree
a. Using boiling water to make pasta or noodles with sauce from a jar	☐	☐
b. Using an oven to heat up store bought frozen or packaged items such as chicken nuggets, French fries, fish	☐	☐
c. Making a salad with already-cut, washed, bagged ingredients and store-bought salad dressing	☐	☐
d. Making something on the stove or oven using mostly scratch or fresh ingredients	☐	☐
e. Using boiling water to make pasta or noodles using fresh ingredients for the sauce	☐	☐
f. Chopped fresh vegetables to make a salad and use homemade salad dressing	☐	☐
g. Use a microwave to defrost frozen meals	☐	☐

Assessment Key	
☐ Easy	☐ Difficult

18. The following questions are about your **thoughts on cooking at home**. Indicate how strongly you agree or disagree with each statement:

	Strongly Disagree	Disagree	Neutral	Agree	Strongly Agree
a. At home, I preferably eat meals that can be prepared quickly	☐	☐	☐	☐	☐
b. The less I have to think about preparing a meal, the better	☐	☐	☐	☐	☐

Cooking Survey for Community Based Health and Needs Assessment

	Strongly Disagree	Disagree	Neutral	Agree	Strongly Agree
c. It is easy to prepare meals	☐	☐	☐	☐	☐
d. I do not like to cook because it takes too much time	☐	☐	☐	☐	☐
e. Cooking meals is a good use of my time	☐	☐	☐	☐	☐

	Strongly Disagree	Disagree	Neutral	Agree	Strongly Agree
f. Cooking at home costs too much	☐	☐	☐	☐	☐
g. Meals made at home are affordable	☐	☐	☐	☐	☐
h. It is not important that I know how to cook	☐	☐	☐	☐	☐
i. It is important to eat the recommended 2 ½ cups of vegetables a day	☐	☐	☐	☐	☐
j. My health is dependent on how and what I eat	☐	☐	☐	☐	☐
k. Making meals at home helps me to eat more healthfully	☐	☐	☐	☐	☐

	Strongly Disagree	Disagree	Neutral	Agree	Strongly Agree
l. Cooking is an important type of relaxation for me	☐	☐	☐	☐	☐
m. I enjoy cooking	☐	☐	☐	☐	☐
n. I enjoy cooking for others	☐	☐	☐	☐	☐
o. Cooking at home is stressful	☐	☐	☐	☐	☐
p. Cooking is frustrating	☐	☐	☐	☐	☐
q. Cooking is fun	☐	☐	☐	☐	☐
r. Cooking is interesting	☐	☐	☐	☐	☐
s. I find cooking tiring	☐	☐	☐	☐	☐

Assessment Key	
☐ Easy	☐ Difficult

19. The following questions are about your **thoughts on your personal cooking habits**. Indicate how strongly you agree or disagree with each statement:

	Strongly Disagree	Disagree	Neutral	Agree	Strongly Agree
a. I like trying new recipes	☐	☐	☐	☐	☐

Cooking Survey for Community Based Health and Needs Assessment

	Strongly Disagree	Disagree	Neutral	Agree	Strongly Agree
b. I feel satisfied when I cook	☐	☐	☐	☐	☐
c. I know how to cook	☐	☐	☐	☐	☐
d. I am confident that whatever I cook will turn out well	☐	☐	☐	☐	☐
e. I identify myself as a cook	☐	☐	☐	☐	☐
f. I know how to create home-made meals that both satisfy my family and do not involve convenience foods.	☐	☐	☐	☐	☐
g. I do not need to use measuring devices (ex: cups, tablespoon, teaspoon, scale) when I cook or create meals from scratch.	☐	☐	☐	☐	☐
h. I know how long certain food items (potatoes, meat, lasagna) will take to cook.	☐	☐	☐	☐	☐
i. I am flexible and can make a meal out of whatever ingredients I have within the house.	☐	☐	☐	☐	☐
j. I can buy healthy foods for my family on a budget.	☐	☐	☐	☐	☐
k. I can cook healthy foods for my family on a budget.	☐	☐	☐	☐	☐
l. I can help my family eat more healthy.	☐	☐	☐	☐	☐
m. When possible, I prefer to buy organic foods (meat, produce)	☐	☐	☐	☐	☐
n. I like to enhance my kitchen to make cooking enjoyable	☐	☐	☐	☐	☐

20. Do you remember the age when you started to cook, if so please state the age.

______

Assessment Key	
☐ Easy	☐ Difficult

Cooking Survey for Community Based Health and Needs Assessment

21. What important factors contributed to the development of your cooking habits.

Assessment Key	
☐ Easy	☐ Difficult

You may select more than one choice.

- ☐ Mother
- ☐ Father
- ☐ Family member (non-parent)
- ☐ Friends/Neighbors
- ☐ T.V. shows
- ☐ Magazines
- ☐ Cookbooks
- ☐ Grandparent (non-parent)
- ☐ Cooking classes
- ☐ Home-economics
- ☐ Self
- ☐ On-line blog/website
- ☐ Online video/apps
- ☐ No important factors

22. Who do you discuss cooking with?

Assessment Key	
☐ Easy	☐ Difficult

- ☐ Family
- ☐ Friends
- ☐ Neighbors
- ☐ No one

23. Do you use any sources of recipes or ideas when you cook? If so, please select all of the choices that you use.

Assessment Key	
☐ Easy	☐ Difficult

- ☐ Cookbooks
- ☐ Magazines
- ☐ Friends
- ☐ Health professional(s)
- ☐ Online blog/website
- ☐ Websites or blogs
- ☐ Family member(s)
- ☐ Neighbors
- ☐ Online apps/videos
- ☐ Myself

24. Do you post messages/photos about cooking on social media (examples Pinterest, Instagram, twitter)?

Between Q. 23 and 24

- ☐ Yes
- ☐ No

25. After reflecting on your cooking practices and habits, how would you rate your diet?

Make last question

Cooking Survey for Community Based Health and Needs Assessment

☐ Excellent ☐ Very Good
☐ Good ☐ Fair ☐ Poor

The following questions represent statements people have made about their food situation.

26. Which of these statements best describes the food eaten in your household in the last 12 months ?

☐ Enough of the kinds of food we want to eat

☐ Enough but not always the kinds of food we want

☐ Sometimes not enough to eat

☐ Often not enough to eat

☐ DK or Refused

27. I (or we) were worried whether my (or our) food would run out before I (or we) got money to buy more." Was that often true, sometimes true, or never true for (you/your household) in the last 12 months?

☐ Often true

☐ Sometimes true

☐ Never true

☐ DK or Refused

28. The food that I (or we) bought just didn't last, and I (or we) didn't have money to get more." Was that often, sometimes, or never true for (you/your household) in the last 12 months?

☐ Often true

☐ Sometimes true

☐ Never true

☐ DK or Refused

29. I (or we) couldn't afford to eat balanced meals. Was that often, sometimes, or never true for you (or your household) in the last 12 months?

- ☐ Often true
- ☐ Sometimes true
- ☐ Never true
- ☐ DK or Refused

30. In the last 12 months from the current month, did you (you or other adults in your household) ever cut the size of your meals or skip meals because there wasn't enough money for food?

- ☐ Yes
- ☐ No (Skip AD1a)
- ☐ Don't know (Skip AD1a)

31. If yes above, how often did this happen—almost every month, some months but not every month, or in only 1 or 2 months?

- ☐ Almost every month
- ☐ Some months but not every month
- ☐ Only 1 or 2 months
- ☐ Don't know

32. In the last 12 months, did you ever eat less than you felt you should because there wasn't enough money for food?

- ☐ Yes
- ☐ No
- ☐ Don't know

33. In the last 12 months, were you every hungry but didn't eat because there wasn't enough money for food?

- ☐ Yes
- ☐ No
- ☐ Don’t know

34. In the last 12 months, did you lose weight because there wasn't enough money for food?

- ☐ Yes
- ☐ No
- ☐ Don’t know

35. In the last 12 months, did you (or other adults in your household) ever not eat for a whole day because there wasn't enough money for food?

- ☐ Yes
- ☐ No (Skip AD5a)
- ☐ Don’t know

36. If you answered yes above, how often did this happen—almost every month, some months but not every month, or in only 1 or 2 months?

- ☐ Almost every month
- ☐ Some months but not every month
- ☐ Only 1 or 2 months
- ☐ Don’t know
