# Supplementary material for: Use of a focus group-based cognitive interview methodology to validate a cooking behavior survey among African-American adults
Source: Front Nutr. 2022 Dec 5;9:1000258. doi: 10.3389/fnut.2022.1000258 (PMC9760831; doi:10.3389/fnut.2022.1000258)
Supplement: Supplementary Table 1 — Survey topics. [file Table_1.DOCX]

**S1 Table.** Survey topics by categories: intrapersonal, interpersonal, cooking practices and selected items or scales from referenced manuscripts.

| **Survey Topics** | **Items or Scales used on Survey**  Exact questions provided in *italics*.  If > 3 questions used or full scale used, please refer to Survey (Supplement Figure) | **Reference** |
| --- | --- | --- |
| **Intrapersonal** |  |  |
| Cooking Self-efficacy | Cooking Self Efficacy for Using Fruits, Vegetables and Seasonings scale (Likert scale) | Michaud, P. |
|  | Cooking Behavior scale (Likert scale) | Michaud, P. |
|  | Cooking Efficacy Scale (Likert scale) | Michaud, P. |
|  | Self-Efficacy for Using Basic Cooking Techniques Scale (Likert scale) | Michaud, P. |
| Attitude and Beliefs | Cooking Attitude scale (Likert scale) | Michaud, P. |
| Food Skills | Food Skills Ability Scale – 12 of 14 items uses | Lavelle, et al |
|  | *I know how to make satisfying meals that both satisfy my family and do not involve convenience foods. (Likert scale with agree response categories)* | Wolfson, et al |
| Cooking Identity | Cooking identity (4 items used) | McGowan, et al |
|  | *I know how to cook (Likert scale with agree response categories)* | Urdapilleta, et al |
| Cooking skills | Cooking Skills Ability - 10 of 12 items used | McGowan, et al |
|  | *I can prepare or cook a healthy meal with only a few ingredients on hand*. *(Likert scale with agree response categories)* | Herbert, et al |
|  | *I am flexible and can make a meal out of whatever ingredients I have within the house.*  *I can buy healthy foods for my family on a budget*  *I can cook healthy foods for my family on a budget*  *I know how long certain food items will take to cook*  *I do not need to use measuring devices when I cook*  *(All 5 questions used Likert scale with agree response categories)* | Ternier and Short |
| Cooking Perception | Cooking Perception Scale (Likert scale) | Wolfson, et al |
| **Interpersonal** |  |  |
| Social/development exposure to cooking | Level of practices (Likert Scale) | Urdapilleta, et al |
| **Cooking Practices** |  |  |
| Cooking practice schedule | Question created from time use survey results showing significance of time of day and day of the week variation for cooking activity.    *What time of the week do you most often cook?* | Daniels, et al |
| Cooking frequency | *During the past 7 days how many times did you or someone in your family cook food for dinner or supper at home?* | FCBS 2009 report |
| Cooking practice time | *How much time do you or someone in your family usually spend on cleaning up after cooking dinner?* | FCBS 2009 report |
