# Supplementary material for: Use of a focus group-based cognitive interview methodology to validate a cooking behavior survey among African-American adults
Source: Front Nutr. 2022 Dec 5;9:1000258. doi: 10.3389/fnut.2022.1000258 (PMC9760831; doi:10.3389/fnut.2022.1000258)
Supplement: Supplementary Table 2 — Question items and illustrative cognitive process. [file Table_2.DOCX]

**Table 2. Participants quoted and illustrated the cognitive process by survey topics and questions with resultant action taken to modify the survey.**

| Original Question Item | Participant Data: Quotes | Participant Data: Theme(s) | Inferred Data: Cognitive Process | Action Taken |
| --- | --- | --- | --- | --- |
|  |  |  |  |  |
| Q. Which person in your household most often prepares main meals During the week?  During the weekend?  Answer choices:  {You, other member of household, don’t know}  Survey Topic: Cooking Practices | " Sometimes my husband does the  breakfast and I do the dinner. Maybe you should separate that and say breakfast and dinner unless I'm missing something.”  "That's true for us too. He does the breakfast." | Clarity of Items  Influence of Individual Roles | Comprehension:  Determination of what information is being asked  Retrieval: Misfit between question terminology and participants’ experiences  Response: Cultural (household) adaptation needed for participant to answer | Made question adaptable to more than one person involved in cooking and . more than one meal. |
| Q. If you are the person cooking in your household during the week or weekend, what time of the week do you most often cook?  Answer choices:  {Weekday morning, afternoon, or evening; Weekend morning, afternoon or evening}  Survey Topic: Cooking Practices | “I was looking for my category and it wasn’t there. So I wrote in, “This question should have a place to write in an answer.”  “Well I was looking for evening after work and it said for weekend”.  “Maybe you could say choose all that apply because I cook weekday evenings sometimes, Saturdays usually. If I’m home, its midday” | Relevance of Items | Comprehension: Determination of what information is being asked  Retrieval: Misfit between question terminology and participants’ experiences  Response: Inadequate response options | Asked about specific day of the week instead of week day and time of the day |
| Q. The following questions describe ways to prepare meals, think about your usual  cooking habits. During the past month how often did you do the following?  Answer choices:  {a.-Prepare meals from basic "scratch" ingredients, such as whole fresh produce,  raw chicken;  b. Prepare meals using convenience items such as bagged salad, prepared mashed  potatoes, pre-shredded vegetables, deli rotisserie chicken  c. Reheat of used leftovers in another meal }  Survey Topic: Cooking Practices | “Prepare meals from basic scratch and ingredients.' I said several times a week but then I had to put a star there saying that wasn't the whole meal, I usually do vegetables, raw chicken...fresh produce yes rice I would do.”  P: “To be honest I had felt like I needed to do something with a because I can clearly see that a is better than b and c.” | Relevance of Items  Social Desirability | Comprehension:  Making sense of the question  Response:  Match between question and response choices;  Social desirability leads to editing of answer  Judgment:  Social desirability | Emphasized in question instructions that components of the meal may contain different preparation methods – scratch, prepared |
| Q. How much time each day do you or someone in your family usually spend on cooking dinner or supper?  Answer choice:  {hours /minutes}  Survey Topic: Cooking Practices | P: "How do you figure out the time it took you to cook cause you're doing so many other things.  P: So, it's hard for you to say it took me an hour or… because then I'm like they would have put something on and then run upstairs and get laundry, run back downstairs. It's not like I'm just sitting there in the kitchen.  P: And I think also sometimes it can depend on if you're using a recipe, about how the recipe you have to marinade. So you could take into consideration the night you might marinate the meat and then the next day you might cook it and then the next day you might cook the sides.  P: "I said I don't know, because she does most of the cooking and then same thing with cleaning up after cooking dinner. I thought about maybe half an hour for that but I actually put 45 minutes “ | Clarity of Items | Comprehension:  Making connections with key term (time) in the question  Retrieval:  Episodic memory, second hand knowledge  Decision/judgment: Combination process averaging  Retrieval:  Recall estimation | Provided specification of time participating in cooking actively |
| Q. Below are several different cooking techniques, please think about which techniques you use at home. If you check I do, then please rate your confidence with using the technique.  Answer choice:  {Deep frying}  Survey Topic: Cooking Skills | " I said I do even though we don't. I know we do."  “I don’t because I don’t want to get burned. That's too for me”  “ That's a guilty question. Yes guilty, we feel guilty that deep frying means that...” | Social Desirability | Response and Judgment: Social desirability | Removed the answer option to choose that participant does a particular technique. Emphasized that if technique is done to mark confidence level |
| Q. Below are several different cooking practices, please think about which practice/habits you use at home. If you check I do use, then please rate your confidence  Answer choices:  { Use knife skills (chopping) in the kitchen to chop vegetables, not necessarily with a cutting board  - Use herbs and spices to flavor food }  Survey Topic: Cooking Skills | Knife skills:  "I put it depends on the sharpness of the knife. Feel more comfortable a dull knife.”  "No, if you're cutting like vegetables some people do them really quickly.”  "without a chopping board that means you're slicing in your hand, right?”  “because after this I found I answered wrong because I do”  “For that one, I answered I do not at all because I was associating what they were saying with the (Participant makes a quick thumping sound).Because I don’t want to lose my fingertips so I don’t do that. But I do cut up and so I would clarify the question with not chopping because I see chopping as (Participant makes quick thumping sound, but i cut up)”  Herbs and spices:  "I use herbs and spices to flavor food. Would that be fresh herbs and spices or...) (P.11) | Clarity of Item | Comprehension:  Misunderstanding of question intent  Determining what information is being asked  Retrieval:  Misfit between question terminology and survey participants’ experiences  Response: Participant adaptation of answer was needed | Added inclusive definition of “knife skills” and specified that dry or fresh herbs and spices are acceptable to report |
| Q. Below are several different food shopping practices, please think about which practice you use when food shopping. If you check I do use, then please rate your confidence with using the shopping practice.  Answer choices:  {Shop with specific meals in mind  Buy food according to season  Buying cheaper cuts of meat to save money}  Survey Topic: Food Shopping Skills | "If you're looking for something specific in mind sometimes you don't find what you're looking for. You know you make a dish and you're looking for a specific herb or meat or something”  "That particular question I said I don't do at all but buying food in season to save money, because I wasn’t sure if for instance if I...like watermelon I love as a fruit. It's seasonal but like dead of winter you know February you probable can find it but it won’t be good.  "Buying cheaper cuts of meat to save money. I would also put under you can buy better carved meat that's on sale  "sometimes people like to go to places like wholesale places...and they’ll buy rotisserie chicken which is a cheap meat. But you're getting fresh cooked meat to save time....Then you see you’re not buying cheaper cuts, you're still getting a good thing but you're getting it at a cheaper price”  “ And at the end of the day, for a lot of our meats now people are going to the organic side. Farm-raised opposed to the way…they better raising them which means it’s going to be a higher cost for people as well. So, when you start doing your survey now, you may want to include the difference with buying organic and buying farm the way they’re what do they call that?” | Relevance of Items | Comprehension: Question wording  Retrieval: Double barreled question and recall issue as behavior is based on knowledge of seasonality  Judgement:  Synthesizing the information retrieved in order to answer the question  Response:  question | Changed response options wording to reflect stated shopping practices, avoided use of language that denoted cheap for economical, as participants associated cheaper with less quality. |
| Q. The following questions are about your thoughts on ways to prepare meals.  Below are some examples of different ways people prepare meals.  Indicate how strongly you agree or disagree that the following activities are cooking:  Answer choices:  {participants given multiple tiered examples of preparation using scratch versus prepared, heated versus no heat combinations]  Survey Topic: Cooking Perception | "This made me think".  : So boiling water to make pasta, cool that's good, then you keep going, or noodles, you're still good, then you say with sauce from a jar.”  "so I guess instead of putting strongly agree or disagree it's either you agree that they're cooking or you disagree.” | Clarity of Items | Comprehension: Determining what information is being asked  Response:  Selecting appropriate scale or response options | Reduced possible choice options for one of the multiple tiered examples |
| Q. What important factors contributed to the development of your cooking habits?  Answer choices:  {mother, father, family member, friends/neighbors, t.v. shows, magazines, cookbooks, on-line blog/website}  Survey Topic: Social Development to Cooking | " I added in my grandmother"  "why is cooking class not a choice?" …"because I had home economics" | Relevance of Items | Response: Inadequate response options | Added in option for specific non-parent family members. |
| Q. Do you use any sources of recipes or ideas when you cook?  {cookbooks, magazines, friends, health professionals, online blog/website, family member(s), neighbors}  Survey Topic: Social Development to Cooking | " I said there should be a space where you could have written in an answer.”  “ I agree with you because I wrote, no not yet."  "Right and I said no as well but because it's not there there's no space for it.” | Relevance of Items | Response: Inadequate response options | Added in other option to reflect open choices |

| Survey Topic | Original Question | Select Participant Quotes | Cognitive Process | Action Taken |
| --- | --- | --- | --- | --- |
| **Cooking Practices** |  |  |  |  |
|  | Q. Which person in your household most often prepares main meals During the week?  During the weekend?  Answer choices:  {You, other member of household, don’t know}[23] | " Sometimes my husband does the  breakfast and I do the dinner. Maybe you should separate that and say breakfast and dinner unless I'm missing something.”  "That's true for us too. He does the breakfast" | Comprehension:  Determination of what information is being asked  Retrieval: Misfit between question terminology and participants’ experiences  Response: Cultural (household) adaptation needed for participant to answer | Made question adaptable to more than one person involved in cooking and . more than one meal. |
|  | Q. If you are the person cooking in your household during the week or weekend, what time of the week do you most often cook?  Answer choices:  {Weekday morning, afternoon, or evening; Weekend morning, afternoon or evening} | “I was looking for my category and it wasn’t there. So I wrote in, “This question should have a place to write in an answer.”  “Well I was looking for evening after work and it said for weekend”.  “Maybe you could say choose all that apply because I cook weekday evenings sometimes, Saturdays usually. If I’m home, its midday” | Comprehension: Determination of what information is being asked  Retrieval: Misfit between question terminology and participants’ experiences  Response: Inadequate response options | Asked about specific day of the week instead of week day and time of the day |
|  | Q. The following questions describe ways to prepare meals, think about your usual  cooking habits. During the past month how often did you do the following?  Answer choices:  {a.-Prepare meals from basic "scratch" ingredients, such as whole fresh produce,  raw chicken;  b. Prepare meals using convenience items such as bagged salad, prepared mashed  potatoes, pre-shredded vegetables, deli rotisserie chicken  c. Reheat of use leftovers in another meal } | “Prepare meals from basic scratch and ingredients.' I said several times a week but then I had to put a star there saying that wasn't the whole meal, I usually do vegetables, raw chicken...fresh produce yes rice I would do.”  P: “To be honest I had felt like I needed to do something with a because I can clearly see that a is better than b and c.” | Comprehension:  Making sense of the question  Response:  Match between question and response choices;  Social desirability leads to editing of answer  Judgment:  Social desirability | Emphasized in question instructions that components of the meal may contain different preparation methods – scratch, prepared |
|  | Q. How much time each day do you or someone in your family usually spend on cooking dinner or supper?  Answer choice:  {hours /minutes} | P: "How do you figure out the time it took you to cook cause you're doing so many other things.  P: So, it's hard for you to say it took me an hour or… because then I'm like they would have put something on and then run upstairs and get laundry, run back downstairs. It's not like I'm just sitting there in the kitchen.  P: And I think also sometimes it can depend on if you're using a recipe, about how the recipe you have to marinade. So you could take into consideration the night you might marinate the meat and then the next day you might cook it and then the next day you might cook the sides.  P: "I said I don't know, because she does most of the cooking and then same thing with cleaning up after cooking dinner. I thought about maybe half an hour for that but I actually put 45 minutes “ | Comprehension:  Making connections with key term (time) in the question  Retrieval:  Episodic memory, second hand knowledge  Decision/judgment: Combination process averaging  Retrieval:  Recall estimation | Provided specification of time participating in cooking actively |
| **Cooking Skills** | Q. Below are several different cooking techniques, please think about which techniques you use at home. If you check I do, then please rate your confidence with using the technique.  Answer choice:  {Deep frying} | " I said I do even though we don't. I know we do."  “I don’t because I don’t want to get burned. That's too for me”  “ That's a guilty question. Yes guilty, we feel guilty that deep frying means that...” | Response and Judgment: Social desirability | Removed the answer option to choose that participant does a particular technique. Emphasized that if technique is done to mark confidence level |
|  | Q. Below are several different cooking practices, please think about which practice/habits you use at home. If you check I do use, then please rate your confidence  Answer choices:  { Use knife skills (chopping) in the kitchen to chop vegetables, not necessarily with a cutting board  - Use herbs and spices to flavor food } | Knife skills:  "I put it depends on the sharpness of the knife. Feel more comfortable a dull knife.”  "No, if you're cutting like vegetables some people do them really quickly.”  "without a chopping board that means you're slicing in your hand, right?”  “because after this I found I answered wrong because I do”  “For that one, I answered I do not at all because I was associating what they were saying with the (Participant makes a quick thumping sound).Because I don’t want to lose my fingertips so I don’t do that. But I do cut up and so I would clarify the question with not chopping because I see chopping as (Participant makes quick thumping sound, but i cut up)”  Herbs and spices:  "I use herbs and spices to flavor food. Would that be fresh herbs and spices or...) (P.11) | Comprehension:  Misunderstanding of question intent  Determining what information is being asked  Retrieval:  Misfit between question terminology and survey participants’ experiences  Response: Participant adaptation of answer was needed | Added inclusive definition of “knife skills” and specified that dry or fresh herbs and spices are acceptable to report |
|  | Q. Below are several different food shopping practices, please think about which practice you use when food shopping. If you check I do use, then please rate your confidence with using the shopping practice.  Answer choices:  {Shop with specific meals in mind  Buy food according to season  Buying cheaper cuts of meat to save money} | "If you're looking for something specific in mind sometimes you don't find what you're looking for. You know you make a dish and you're looking for a specific herb or meat or something”  "That particular question I said I don't do at all but buying food in season to save money, because I wasn’t sure if for instance if I...like watermelon I love as a fruit. It's seasonal but like dead of winter you know February you probable can find it but it won’t be good.  "Buying cheaper cuts of meat to save money. I would also put under you can buy better carved meat that's on sale  "sometimes people like to go to places like wholesale places...and they’ll buy rotisserie chicken which is a cheap meat. But you're getting fresh cooked meat to save time....Then you see you’re not buying cheaper cuts, you're still getting a good thing but you're getting it at a cheaper price | Comprehension: Question wording  Retrieval: Double barreled question and recall issue as behavior is based on knowledge of seasonality  Judgement:  Synthesizing the information retrieved in order to answer the question  Response:  question | Changed response options wording to reflect stated shopping practices, avoided use of language that denoted cheap for economical, as participants associated cheaper with less quality. |
| **Cooking Perception**  **(How a person defines cooking)** | Q. The following questions are about your thoughts on ways to prepare meals.  Below are some examples of different ways people prepare meals.  Indicate how strongly you agree or disagree that the following activities are cooking:  Answer choices:  {participants given multiple tiered examples of preparation using scratch versus prepared, heated versus no heat combinations] | "This made me think".  : So boiling water to make pasta, cool that's good, then you keep going, or noodles, you're still good, then you say with sauce from a jar.”  "so I guess instead of putting strongly agree or disagree it's either you agree that they're cooking or you disagree.” | Comprehension: Determining what information is being asked  Response:  Selecting appropriate scale or response options | Reduced possible choice options for one of the multiple tiered examples |
| **Social/development exposure to cooking** | Q. What important factors contributed to the development of your cooking habits?  Answer choices:  {mother, father, family member, friends/neighbors, t.v. shows, magazines, cookbooks, on-line blog/website} | " I added in my grandmother"  "why is cooking class not a choice?" …"because I had home economics" | Response: Inadequate response options | Added in option for specific non-parent family members. |
|  | Q. Do you use any sources of recipes or ideas when you cook?  {cookbooks, magazines, friends, health professionals, online blog/website, family member(s), neighbors} | " I said there should be a space where you could have written in an answer.”  “ I agree with you because I wrote, no not yet."  "Right and I said no as well but because it's not there there's no space for it.” | Response: Inadequate response options | Added in other option to reflect open choices |
